# Editorial Comment: Is the Effectiveness of Self-Visualization During Flexible Cystoscopy Gender-Dependent in Patients with no Previous Cystoscopy History?

**DOI:** 10.1590/S1677-5538.IBJU.2025.0061

**Published:** 2025-03-20

**Authors:** Hinpetch Daungsupawong, Viroj Wiwanitkit

**Affiliations:** 1 Private Academic Consultant Phonhong Laos Private Academic Consultant, Phonhong, Laos; 2 Chandigarh University University Centre for Research & Development Department of Pharmaceutical Sciences Mohali Punjab India University Centre for Research & Development Department of Pharmaceutical Sciences, Chandigarh University, Mohali, Punjab, India

To the editor,

We would like to comment on "Is the Effectiveness of Self-Visualization During Flexible Cystoscopy Gender-Dependent in Patients with no Previous Cystoscopy History? A Prospective Randomized Study (1)". This study looks into the effects of real-time self-visualization (SV) during flexible cystoscopy (FC) on discomfort, anxiety, patient satisfaction, and willingness to repeat the procedure. Male patients in the SV group had considerably lower pain scores and anxiety levels than those in the non-SV group, as well as higher satisfaction and readiness for future cystoscopy. However, no significant variations in pain outcomes were detected across groups among female patients. Although this study provides fascinating insights, there are several questions about its design and approach.

The biggest limitation of this study is the possibility of gender bias. According to the study, the SV group included an equal number of male and female patients, however the findings indicated that the SV intervention had a stronger effect on pain reduction in men than in women. This raises the question of whether gender-related variables such as pain perception, anxiety levels, and tolerance for medical procedures were not sufficiently controlled or examined. Differences in baseline features and psychological aspects between male and female patients may influence the results, but these variables were not controlled for in this investigation. Furthermore, selection bias may have been introduced by the randomization strategy, which assigned male patients to the SV and non-SV groups sequentially, as well as women. Although the 1:1 ratio was maintained, further randomization may not have adequately compensated for confounding variables.

The statistical analysis in this study may have been insufficient to determine the significance of pain scores in female patients. Although the pain difference among women was not statistically significant, the tiny effect size could have resulted in a type II error due to sample size or insufficient statistical power for this subgroup. The lack of multivariate analysis to account for potential confounding factors, such as prior medical procedures or experience with anxiety disorders, further limits the study's ability to make definite results.

Future research should include bigger and more diverse groups with thorough stratification for confounding factors (e.g., gender, baseline anxiety level, medical history) to improve the validity of the results. Furthermore, a longitudinal study assessing the long-term effects of self-visualization on anxiety, pain tolerance, and patient satisfaction over numerous cystoscopy sessions could give additional evidence of its efficacy. Alternative approaches that combine self-visualization with other pain-relieving techniques may also provide useful insights for enhancing the patient experience during cystoscopy. Finally, blinded study design may reduce bias in outcome evaluation, particularly for subjective variables like pain and satisfaction.
